# Major parasitic diseases of poverty in mainland China: perspectives for better control

**DOI:** 10.1186/s40249-016-0159-0

**Published:** 2016-08-01

**Authors:** Jin-Lei Wang, Ting-Ting Li, Si-Yang Huang, Wei Cong, Xing-Quan Zhu

**Affiliations:** State Key Laboratory of Veterinary Etiological Biology, Key Laboratory of Veterinary Parasitology of Gansu Province, Lanzhou Veterinary Research Institute, Chinese Academy of Agricultural Sciences, Lanzhou, Gansu Province 730046 People’s Republic of China

**Keywords:** China, Poverty, Parasitic diseases of poverty, Epidemic characteristics, Prevention, Control, Elimination

## Abstract

Significant progress has been made in the prevention, control, and elimination of human parasitic diseases in China in the past 60 years. However, parasitic diseases of poverty remain major causes of morbidity and mortality, and inflict enormous economic costs on societies.

In this article, we review the prevalence rates, geographical distributions, epidemic characteristics, risk factors, and clinical manifestations of parasitic diseases of poverty listed in the first issue of the journal *Infectious Diseases of Poverty* on 25 October 2012. We also address the challenges facing control of parasitic diseases of poverty and provide suggestions for better control.

**Electronic supplementary material:**

The online version of this article (doi:10.1186/s40249-016-0159-0) contains supplementary material, which is available to authorized users.

## Multilingual abstracts

Please see Additional file 1 for translations of the abstract into the six official working languages of the United Nations.

## Background

Infectious diseases of poverty (IDoPs) are a series of diseases closely related to poverty, which are mainly prevalent in the least developed countries and regions of the world [1–3]. Most IDoPs are neglected tropical diseases, causing disabling chronic infections, and even death [2]. In addition, IDoPs can result in huge economic losses and make it more difficult for poor people to improve their quality of life and social status [3].

It has been estimated that IDoPs affect three billion people worldwide, and they kill almost nine million people each year, many of them are children under five [3]. Factors such as climate change and population migration have accelerated the spread of these diseases, causing health and socioeconomic problems globally. It is a vicious cycle: poverty due to endemic IDoPs depresses economies in the affected communities by reducing people’s ability to work. This in turn renders the poor less able to pay for treatment and also aggravates transmission of IDoPs [2–6].

Although tremendous achievements have been made in fighting IDoPs in China in the past 60 years, the sheer scale of the task means that the country still accounts for a large percentage of the global burden of disease due to IDoPs. For example, schistosomiasis still affects at least a hundred thousand people in China, and up to 90 % of the world’s burden of alveolar echinococcosis is attributed to China, where about 86 million people are at risk [7–9]. Of the 15 million people who are infected with *Clonorchis sinensis* globally, over 85 % live in China [7–9].

In this review, our main objectives are to describe the prevalence rates, geographical distributions, epidemic characteristics, risk factors, and clinical manifestations of parasitic diseases of poverty listed in first issue of the journal *Infectious Diseases of Poverty* on 25 October 2012, which have caused extensive damage in China (see Table 1) [1]. We also discuss the current challenges and strategies for controlling these diseases.

**Table 1:** Key characteristics of parasitic diseases of poverty in China

Disease	Parasite	Definitive host	Intermediate host	Factors relating to infection^a^	Clinical manifestations	DALYs^b^ (thousands)
Vector-borne diseases						
Malaria [10–13]	*Plasmodium* spp.	Humans and other animals	Mosquito	Children, pregnant women, humble house, lack of bed nets, immigrants from epidemic regions, occupation dependent exposure to mosquitoes	Fever, headache, shock, jaundice, abnormal bleeding, nausea, vomiting, diarrhea, anemia, hepatosplenomegaly	82 685
Leishmaniasis [26–28]	*Leishmania* spp.	Humans and other mammals	Sandfly	Children, the older, males, dog ownership, herdsman, humble house, sleeping outside, occupation dependent exposure to sandfly	CL: Skin papules, plaque, ulcer and nodular prurigo ML: Edema and erythema on nose, nasal stuffiness or bleeding, mucosal lesions VL: Fever, weight loss, anemia, hepatosplenomegaly	3 317
Lymphatic filariasis [31–33]	*Wuchereria bancrofti* *Brugia malayi* *Brugia timori*	Humans	Mosquito	Humble house, lack of bed nets, occupation dependent exposure to mosquitoes	Lymphangitis, lymphnoditis, lymphoedema, elephantedema hydrocele, chyluria	2 775
Snail-borne diseases						
Schistosomiasis [35–37]	*Schistosoma* spp.	Humans and other mammals	*Oncomelania hupensis*	Males, high frequency of water contact, occupation dependent exposure to snails, snails related practices	Fever, headache, abdominal pain, hematuria, anemia, bloody stool, hepatosplenomegaly, colonic tumoroid proliferation, ascites, hydronephrosis, dwarfism, megalosplenia	3 309
Clonorchiasis [42–44]	*Clonorchis sinensis*	Humans and other mammals	Water snails, fish and shrimps	Males, the older, high frequency of eating of raw or undercooked freshwater fish	Inappetence, abdominal pain, gallstone, jaundice, anemia, hepatosplenomegaly, pyogenic cholangitis, cholecystitis	275
Paragonimiasis [54]	*Paragonimus* spp.	Humans, cats, dogs and other carnivores	Water snails and crustaceans	Children, high frequency of eating of raw or undercooked freshwater crabs	Chronic cough, chest pain, hemoptysis, pleurisy, dyspnea, abdominal pain, epilepsy	197
Fascioliasis [57–59]	*Fasciola gigantica* *Fasciola hepatica*	Humans and other mammals	Water snails	Children, females, high frequency of eating of raw vegetables or untreated water and contact with ruminants	Fever, anemia, hepatic lesions and fibrosis, hepatomegaly, jaundice, cholangitis, cholecystitis	35
Soil-transmitted helminthiasis						
Ascariasis	*Ascaris lumbricoides*	Humans	-	Ascariasis and trichuriasis: school-aged children Hookworm: the older and farmers	Inappetence, undernutrition, abdominal pain, diarrhea, anemia, growth and cognitive deficits	1 315
Trichuriasis	*Trichuris trichiura*	Humans	-			638
Hookworm [63–66]	*Ancylostoma duodenale Necator americanus*	Humans	-			3 231
Enterobiasis [66, 70, 72]	*Enterobius vermicularis*	Humans	-	School-aged children, crowded	Inappetence, restlessness, perianal pruritus and discomfort, insomnia, irritability, growth and cognitive deficits	-
Zoonotic diseases						
Taeniasis/Cysticercosis [73, 74, 76]	*Taenia solium* *Taenia saginata Taenia asiatica*	Humans	Humans, pig and cattle	Pigs related practices, high frequency of eating of raw pork	Headaches, ocular disorders, epilepsy, seizure, neurological symptoms	503
Echinococcosis [77–80, 84–86]	*Echinococcus granulosus* *Echinococcus multilocularis*	Dogs and wild canids	Humans and other animals	Females, the older, herdsmen slaughter and viscera disposal practices, dog related practices	AE: Tumour-like multi-vesicular CE: Unilocular fluid-filled bladders	144
Water-borne diseases						
Cryptosporidiosis [88–90]	*Cryptosporidium* spp.	Humans and other animals	-	Children, immunocompromised individuals, poor water treatment	Diarrhea, growth deficits, malnutrition, weight loss	8 372
Giardiasis [95]	*Giardia* spp.	Humans and other animals	-	Children, poor water treatment	Diarrhea, malnutrition, growth deficits, weight loss	-
Toxoplasmosis [98–101]	*Toxoplasma gondii*	Felids	Humans and other animals	The older, cancer patients, immunocompromised individuals, cat related practices	Blindness, mental deficiency, encephalitis, stillbirths, abortion	-
Outbreak parasitic diseases						
Angiostrongyliasis [104–107]	*Angiostrongylus cantonensis*	Rats	Snails and slugs	High frequency of eating of raw or undercooked snails	Eosinophilic meningitis, headache, somnolence, ocular angiostrongyliasis	-

^a^Poverty, poor sanitation and inadequate hygiene and poor knowledge of, attitudes towards and practice relating to parasites are also the predisposing factors to these diseases. ^b^Source: WHO, Global Burden of Diseases 2010 [42, 130]

## Review

### Vector-borne parasitic diseases

#### Malaria

Malaria, one of the most threatening diseases worldwide, is endemic in over 100 developing countries, with about 58 % of deaths due to malaria occurring in poor communities [10–13]. Five species of the genus *Plasmodium* commonly infect human beings. *Plasmodium falciparum* and *P. vivax* cause the majority of infections, with most malaria deaths caused by *P. falciparum*. Both of these species may cause abortion and intrauterine growth retardation if infection occurs during pregnancy. The species *P. ovale*, *P. malariae*, and *P. knowlesi* can also infect humans but are uncommon, and do not manifest in serious symptoms [10–13].

Although significant progress has been made in reducing the malaria burden, there were still 198 million malaria cases worldwide in 2013, causing 584 000 deaths [14–16]. In China, malaria, mainly due to *P. falciparum* and *P. vivax* can be traced back 4 000 years. Before the foundation of the People’s Republic of China, malaria threatened 75 % of the Chinese population: over 30 million cases were recorded annually with about a 1 % mortality rate. Since 1949, the overall burden of malaria has been markedly reduced and endemic regions have greatly reduced in area (see Table 2) [17–22]. However, this ancient disease still represents a serious public health challenge in China, and some new problems are emerging. Before 2012, *P. vivax* was the major species of malaria parasite in China. Despite the lower mortality caused by this species, it is a major cause of morbidity. After the National Malaria Elimination Programme was launched in 2010, the incidence of locally transmitted *P. vivax* malaria has declined, but numbers of imported *P. vivax* and *P. falciparum* malaria cases have increased significantly due to the large number of migrant workers and travelers coming into China. *P. falciparum* has become the major imported species of malaria (see Table 2) [17–23]. Antimalarial drug-resistant *Plasmodium* spp. emerged in some endemic areas and global warming has lead to an expansion of the habitats of mosquitoes [24]. These factors are making it more challenging to achieve the goal of eliminating malaria by 2020 throughout the entire country, but with continued efforts and new surveillance-response systems, the goal can be achieved.

**Table 2:** The characteristics of malaria in China from 2011 to 2014 [18–21]

Year	Number of malaria cases	Number of deaths	Annual incidence	The proportion of endemic counties	The proportion of laboratory confirmed cases	The proportion of foreign imported cases	Major malaria endemic province
2011	4 479 (3 658 laboratory confirmed cases and 821 clinically diagnosed cases)	33	0.0334/10 000	27.4 % (782/2 856)	*P. falciparum*: 40.2 % *P. vivax*: 56.7 % *P. ovale* or *P. malaride:* 1.9 % Mixed infection: 1.1 %	66.4 %	Anhui 40.0 % Yunnan 25.8 % Henan 12.6 % Guizhou 10.4 % Hubei 6.1 %
2012	2 718 (2 599 laboratory confirmed cases and 119 clinically diagnosed cases)	15	0.0202/10 000	21.7 % (620/2 853)	*P. falciparum*: 54.6 % *P. vivax*: 41.6 % *P. ovale* or *P. malaride*: 2.1 % Mixed infection: 1.7 %	91.0 %	Yunnan 31.4 % Guangxi 8.1 % Jiangsu 7.3 % Hunan 5.8 % Sichuan 5.7 %
2013	4 128 (4 087 laboratory confirmed cases and 41 clinically diagnosed cases)	23	0.0305/10 000	21.2 % (605/2 852)	*P. falciparum*: 71.2 % *P. vivax*: 22.8 % *P. ovale* or *P. malaride*: 4.5 % Mixed infection: 1.6 %	97.9 %	Guangxi 30.3 % Yunnan 14.0 % Jiangsu 8.3 % Sichuan 5.8 % Zhejiang 5.0 %
2014	3 078 (3 057 laboratory confirmed cases and 21 clinically diagnosed cases)	25	0.0226/10 000	23.8 % (680/2 853)	*P. falciparum*: 61.6 % *P. vivax*: 27.7 % *P. ovale* or *P. malaride*: 9.3 % Mixed infection: 1.4 %	98.1 %	Yunnan 17.3 % Jiangsu 11.5 % Sichuan 8.6 % Henan 7.0 % Zhejiang 7.0 %

#### Leishmaniasis

Leishmaniasis is highly prevalent in poor countries in Southeast Asia, East Africa, and Latin America. The disease is present in 88 countries, resulting in 1.5–2 million new cases each year [25]. The three different forms of leishmaniasis are cutaneous leishmaniasis (CL), mucosal leishmaniasis (ML), and visceral leishmaniasis (VL) [26–28]. VL, the most severe form, is caused by *Leishmania donovani* or *L. infantum*, and falls into three different epidemiological types in China: the anthroponotic type, the zoonotic mountain type, and the zoonotic desert type [28]. It was once highly prevalent and rampant in areas north and northwest of the Yangtze River, especially in the rural regions of Shandong, Jiangsu, Anhui, and Henan provinces. In 1951, about 530 000 VL cases occurred across more than 650 counties in at least 16 provinces/autonomous regions/municipalities (P/A/Ms) in China. National control programs undertaken in the 1950s aimed at exterminating the sandfly vector, curing infected dogs, and treating patients. Since then, the disease has been effectively controlled and almost eliminated in eastern and northern China. The number of VL cases decreased to 360 in 1990, with some sporadic cases reported in six provinces in the central and western China: Xinjiang Uygur and Inner Mongolia Autonomous Regions, and Gansu, Sichuan, Shaanxi, and Shanxi provinces. However, transmission in these provinces was never completely interrupted and the number of VL cases has increased from 2003 to 2009. The number of areas of transmission has expanded in recent years due to population movement and ecological changes. This might explain the outbreak of VL in Jiashi county, Xinjiang Uygur in 2009 [28, 29]. A total of 3601 cases were officially reported between 2004 and 2014 in China, varying from 158 to 509 cases per year. There was a sharp increase in 2008 and 2009, with an incidence of 0.0169/100 000 in 2009 [30]. More than 97 % of these cases were concentrated in Xinjiang Uygur, Gansu, and Sichuan, where reservoirs of *Leishmania* and sandfly vectors remain common [30].

#### Lymphatic filariasis

Lymphatic filariasis is caused by three closely-related filarial nematodes: *Wuchereria bancrofti*, *Brugia malayi*, and *B. timori*. This disease affects more than 120 million people worldwide, about 40 million of them are disabled and disfigured [31–33]. Sixty years ago, lymphatic filariasis was widely prevalent in mainland China, with 31 million cases and 330 million people at risk living in 864 endemic counties in 16 P/A/Ms, mainly in southeastern China. Bancroftian filariasis accounted for about two-thirds of these cases (~22 million) and nine million were Brugian filariasis. Since then, excellent control measures have been introduced, including treatment of infected individuals with diethylcarbamazine (DEC) and the distribution of DEC-fortified salt. The latter measure, in particular, has been very effective for the control and treatment of this disease. By 1980, basic elimination of filariasis was achieved in many endemic counties. By 1994, all 864 endemic counties had reached the basic elimination criteria and reduced the microfilaremia rate to below 1 %. After decades of efforts in control and surveillance of filariasis, China became the first developing country to eliminate lymphatic filariasis in 2007, as verified by the World Health Organization [34]. However, there are still about 400 000 chronic cases resulting from past infections in China, and there is threat of imported infection. Therefore, China should strengthen its surveillance-response system for this disease and provide medical care to patients to overcome these challenges [7].

### Snail-borne parasitic diseases

#### Schistosomiasis

Schistosomiasis causes substantial morbidity and mortality, enforcing a cycle of poverty, especially in already poor rural communities. Three major species (*Schistosoma japonicum*, *S. haematobium*, and *S. mansoni*) can infect humans, with at least 230 million people in approximately 76 developing countries being afflicted [35, 36]. In China, schistosomiasis due to *S. japonicum*, once a widespread serious disease, has been recorded over the past 2 100 years. In the 1950s, *S. japonicum* was endemic in 12 P/A/Ms, with around 11.6 million people infected and more than 100 million people at risk of infection. It was estimated that over 1.2 million cattle were infected and that suitable habitat for the snail intermediate host (*Oncomelania hupensis*) covered an area of 14.3 billion square meters. After the foundation of the People’s Republic of China, the government finally comprehended the enormous social and economic losses due to *S. japonicum* and adopted a series of prevention and control strategies against the disease. These can be separated into three phases. During the first phase (1950s to early 1980s), snail control was the strategy used to limit transmission of schistosomiasis. Environmental modifications and use of molluscicides in snail habitats significantly reduced the prevalence of the disease. In the second phase (mid-1980s to 2003), large-scale chemotherapy and morbidity control were implemented. Assisted by a World Bank loan, the widespread mass administration of praziquantel reduced the number of schistosomiasis cases from about 1 522 100 in 1989 to 865 000 in 1995. During the third phase (2004 until now), an integrated strategy is being used to reduce potential sources of infection. The intention is to prevent the spread of *S. japonicum* eggs from the feces of cattle and humans to snails. By 2008, the prevalence rate, both in humans and cattle, had decreased to less than 5 % in all endemic counties [37–39]. The number of schistosomiasis cases fell from 842 525 in 2004 to 115,614 in 2014. The number of acute cases reduced from 816 in 2004 to two in 2014, and the extent of suitable habitat for *O. hupensis* snails was reduced from 7 billion square meters in 2004 to 3.64 billion square meters in 2014 [40, 41]. Some P/A/Ms, including Guangxi Zhuang Autonomous Region and Shanghai, Zhejiang, Guangdong, and Fujian provinces, have now achieved the national criteria for elimination. However, schistosomiasis is still endemic in Anhui, Jiangxi, Hunan, Sichuan, and Yunnan provinces. Factors including global warning and population movements have the potential to cause the reemergence of schistosomiasis [40, 41]. Challenges such as lack of effective tools for snail control, and the effect of climate and ecosystem changes remain to be overcome if the goal of schistosomiasis elimination by 2020 is to be achieved.

#### Clonorchiasis

Clonorchiasis caused by *Clonorchis sinensis* is mainly distributed in East Asia and leads to a significant disease burden, with approximately 15 million people infected [42, 43]. The most severe complication is cholangiocarcinoma [44]. In 2009, the International Agency of Cancer Research classified *C. sinensis* as a group 1 carcinogen [45]. China accounted for the largest proportion of clonorchiasis cases, with over 13 million people infected in 2004 [43, 46]*.* From 1988 to 1992 and 2001 to 2004, two national surveys were conducted, respectively, and indicated that the prevalence of clonorchiasis has increased from 0.37 % in 30 P/A/Ms in 1992 to 0.58 % in 31 P/A/Ms in 2004 [47, 48]. The infection rate and intensity of infection were higher in males than in females and were reported to increase with age, peaking at 50–59 years in 2004 [48]. There are two major epidemic zones in China. The first is in northeastern China and includes Heilongjiang, Jilin, and Liaoning provinces. In Heilongjiang, a study of 4951 clinically suspected outpatients between 2009 and 2012 confirmed that 25.93 % had clonorchiasis, with the highest prevalence (34.25 % [437/1 276]) recorded in 2012 [49]. The second zone includes Guangdong and Guangxi Zhuang in Southern China [42, 43, 46]. Guangdong had the highest prevalence (16.4 %) followed by Guangxi Zhuang (9.8 %) in 2004 [48]. Surveys conducted in Hengxian County, Guangxi Zhuang, between 1989 and 2011 and in Guangzhou city, Guangdong between 2006 and 2012 found a trend towards increasing infection rates and intensities [50, 51]. Many animals serve as reservoir hosts for *C. sinensis* with potentially high prevalence rates: 20.5 % in dogs and 41.8 % in cats recorded in Guangdong in 2008 [52]. The Chinese government is well aware of the importance of this disease and has take some measures to control this disease such as the prevention of contamination of fish ponds and aquaculture systems by faeces, the control of snails, and the implementation of education campaigns [53].

#### Paragonimiasis

Paragonimiasis, caused by lung flukes of the genus *Paragonimus*, is an important food-borne zoonosis. At least 10 of the >30 named *Paragonimus* species are known to infect humans [42, 54]. About 40 % of the 56 million food-borne trematode infections worldwide are caused by *Paragonimus* spp.. The disease is endemic in Asia, Africa, and the Americas [42, 54], but most cases (~90 %) are distributed in Asia, and the majority of those are in China [54]. The second national survey, conducted in 2004, showed that the overall prevalence of paragonimiasis in China was about 1.7 %, indicating that about 22 million people were infected. In that survey, Shanghai and Chongqing cities had the highest infection rates (about 5.1 % and 4.1 %, respectively) [48]. In China, human paragonimiasis is mainly caused by *Paragonimus skrjabini* and *P. westermani*, which use freshwater snails as their first intermediate hosts. Many species of crabs and crayfish can act as second intermediate hosts. The prevalence of *Paragonimus* in the first and second intermediate hosts can be high [54]. One small-scale survey conducted in Dazhou city, Sichuan in 2013 found a 22.7 % prevalence of metacercariae in crabs [55]. Although there have been no recent large-scale surveys conducted on the disease in humans, some small-scale surveys have indicated that prevalence of paragonimiasis has not greatly diminished in the country: e.g. rates of 3.6 % (30/840) in Dazhou and 2.6 % (35/1,373) in Xingshan county, Hubei province were recorded in 2012 [55, 56].

#### Fascioliasis

Fascioliasis is caused by *Fasciola gigantica* and *F. hepatica.* The distribution of these species is largely dictated by the geographical ranges of the freshwater snail species that act as intermediate hosts. *Fasciola hepatica* has a near-global geographical distribution, whereas *F. gigantic* occurs in tropical regions mainly in Africa and Asia [42]. Fascioliasis in livestock has always been well recognized because of the enormous economic costs incurred due to the disease [57]. It has been estimated that about 700 million production animals are at risk of infection, leading to an economic loss of over $2 billion per year. However, human fascioliasis has been markedly neglected, with only about 2 500 human cases reported before the 1990s [57–59]. In the last 20 years, due to climate and global changes, in some regions fascioliasis is emerging or remerging in humans and animals, and several outbreaks of human fascioliasis have been reported. It has been estimated that millions of people, mainly in low-income countries, are infected, with an additional 180 million at risk of infection [57].

In China, *F. gigantica* is endemic mainly in tropical and subtropical regions such as Guangxi Zhuang, Guangdong, and Yunnan, whereas *F. hepatica* occurs throughout the country. Only 44 human fascioliasis cases were reported before the 1990s [60]. According to the first national survey conducted in 1992, 120 000 people were infected, with regional prevalence rates ranging from 0.002 % to 0.017 %. Prevalence rates were highest in Gansu [47]. Sporadic human cases and outbreaks are reported from time to time. For example, in Yunnan, 15 cases of *F. hepatica* infection were reported in 2005, and 29 cases of *F. gigantica* infection in 2012 [61]. High prevalence rates have been reported in ruminants. For example, in Ili Kazak Autonomous prefecture, Xinjiang Uygur, the prevalence of *Fasciola* infection in sheep was 42.7 % (983/2,300) in 2011 [62]. In Yunnan, 28.6 % of cattle and 26.0 % of goats were infected with fascioliasis in 2012 [61]. Limited surveys about the prevalence of the disease in humans and animals have hindered the design of more effective control and surveillance systems in the country.

### Soil-transmitted helminths

Soil-transmitted helminths (STHs), mainly ascariasis, trichuriasis, and hookworm infections, are nonlinearly correlated with poverty and are highly prevalent in developing countries, causing immense disease burdens [63–66]. Over five billion people are at risk and at least one billion are infected with at least one STH species globally. Around 300 million people suffer from severe morbidity. Ascariasis and trichuriasis, caused by the ingestion of infective eggs of the roundworm *Ascaris lumbricoides* and the whipworm *Trichuris trichiura,* affect more than 800 million and 600 million people worldwide, respectively. Hookworm infections, acquired by active penetration of the skin by worm larvae in the soil, are caused by *Ancylostoma duodenale* and *Necator americanus*. Over 700 million people are infected and over 130 000 deaths are caused annually from these infections [63–66]. The highest morbidity rates occur in the rural poor and school-aged children.

In China, human infections with STHs were common in the past. However, after years of efforts using control measures such as chemotherapy, sanitation, and health promotion, the prevalence has decreased significantly. The first national survey, conducted in 1992, revealed an overall prevalence of 47.0 %, 18.8 %, and 17.2 % for ascariasis, trichuriasis, and hookworm, respectively. The second national survey, conducted in 2004, found that the respective prevalence rates were 12.7 %, 4.6 %, and 6.1 %, a reduction of about 407 million infections [47, 48]. Prevalence rates and intensities of STH infections continue to decline each year. In 2010, the respective prevalence rates were further reduced to 6.8 %, 1.8 %, and 3.7 % [67]. However, some provinces still had high prevalence of STH infection such as 40.8 % in Hainan, 34.6 % in Guizhou, and 30.6 % in Sichuan in 2010 [67]. The prevalence rates in some poor rural areas in these provinces are higher. For example, a large-scale survey of 2179 children aged 9–11 years living in impoverished rural areas in Guizhou province showed that 42.0 % were infected with at least one STH species in 2013 [68]. Another survey revealed that the prevalence rates of STH infections in children of school age and below in poor rural areas of Guizhou and Sichuan was 21.2 % and 22.9 % in 2013, respectively [69]. Therefore, STH infections are still a significant health problem for both children and adults living in poor rural areas.

#### Enterobiasis

In addition to the three major STHs discussed above, enterobiasis, caused by *Enterobius vermicularis*, is one of the most highly prevalent parasitic diseases in children. *Enterobius vermicularis* is the oldest and most widely distributed pinworm affecting about 400 million people worldwide (with 4–28 % of children infected globally) [66, 70]. According to national surveys conducted in China, the prevalence of *E. vermicularis* infection in children has decreased from 23.6 % in 1992 to 10.3 % in 2004, and further to 6.6 % in 2010 [47, 48, 71]. Despite overall reductions at the national level, however, infection rates in some endemic areas have actually increased. The prevalence rate rose to 46.1 % in Guangdong in 2010 [70]. A recent survey of 802 children conducted in Gaozhou city, Guangdong in 2011 showed a *E. vermicularis* prevalence of 54.9 % [72].

### Zoonotic parasitic diseases

#### Taeniasis and cysticercosis

Taeniasis and cysticercosis affect millions of people and incur significant economic costs primarily in developing countries. Humans, the obligate definitive hosts for the species *Taenia solium*, *T. asiatica*, and *T. saginata*, harbor the adult tapeworm in the small intestine after consumption of raw or undercooked pork or beef contaminated with cysticerci. The disease usually manifests in symptoms such as mild abdominal pain and can even be asymptomatic [73, 74]. However, people harboring tapeworms shed eggs in their feces, which is a potential source of infection for animals and humans. Human cysticercosis is caused by the accidental ingestion of *T. solium* eggs or via autoinfection. Infection of the human central nervous system leads to neurocysticercosis, which is considered to be the most frequent cause of acquired epilepsy and seizures worldwide. At least 30 million people have symptomatic neurocysticercosis, with 50,000 deaths recorded each year [73, 74].

In China, human taeniasis is caused by the three *Taenia* species listed above. Over 550 000 people are infected. Human cysticercosis is heavily endemic in China because of the consumption of traditional pork products [48]. According to two national surveys, the prevalence rates of taeniasis and cysticercosis have increased significantly. Taeniasis increased from 0.18 % in 1992 to 0.28 % in 2004, and cysticercosis from 0.01 % in 1992 to 0.58 % in 2004. The Tibet Autonomous Region had the highest prevalence of taeniasis (19.2 %) and Shaanxi had the highest prevalence of cysticercosis (3.4 %) in 2004 [47, 48]. Although in recent years, only small-scale surveys of these infections have been conducted, and the findings indicated that taeniasis and cysticercosis are still endemic in China. One survey in 2007 showed that the prevalence rates of taeniasis in Yajiang county, Sichuan, and Ming county, Gansu were 6.4 % (73/1,137) and 4.1 % (27/652), respectively, and the prevalence of cysticercosis in Ming county was 2.6 % (17/652) [75]. Another survey conducted in Danba County, Sichuan from 2008 to 2014 found a prevalence of 2.3 % (23/1 013) for cysticercosis. Tibetans have a habit of eating uncooked meat, leading to a high incidence of cysticercosis [76]. Some measures have been taken to control these diseases such as toilet amelioration, inspection of meat in the markets, and introduction of the intensive rearing systems.

#### Echinococcosis

Human echinococcosis is increasingly considered to be one of the most challenging problems for human and animal health globally. *Echinococcus granulosus* and *E. multilocularis*, the two major species infecting humans, cause human cystic echinococcosis (CE) and alveolar echinococcosis (AE), respectively. Geographically, CE has a worldwide distribution, while AE is rare and limited to a few regions, located at relatively high latitudes in the northern hemisphere [77–79]. The mortality rate of AE is usually higher than that of CE. Globally, it has been estimated that over three million people are infected with *Echinococcus* [80, 81]. Echinococcosis is heavily endemic in China, with 0.38 million people infected, and accounting for 40 % of the global CE disability-adjusted life years (DALYs) and more than 90 % of global AE DALYs [80, 81]. Recent epidemiological studies indicate that echinococcosis is widespread in the central and western regions of China including Xinjiang Uygur, Inner Mongolia, and Ningxia Hui Autonomous Regions, and Qinghai, Gansu, and Sichuan provinces, and that prevalence rates appear to have gradually risen in recent years. In China, human CE cases are responsible for more than 90 % of echinococcosis infections, with AE cases forming the remainder. The latter are spatially correlated with climate and landscape characteristics. The prevalence of human AE was found to decrease with the ratio of forests and increase with the ratio of alpine meadows [82]. Seroepidemiological surveys conducted in highly endemic areas of western China have indicated a prevalence of echinococcosis of 5–30 % in humans, 5–67 % in dogs, 26–82 % in sheep, and 38–78 % in yaks [83–87].

### Waterborne parasitic diseases

#### Cryptosporidiosis

Cryptosporidiosis, caused mainly by *Cryptosporidium hominis* and *C. parvum*, is one of the most common causes of diarrhea and gastroenteritis in humans and domestic animals [88–90]. Although globally distributed, the prevalence of cryptosporidiosis in immunocompetent individuals in low-income countries (5–10 %) is higher than that in advanced economies (1 %). The greatest burden of cryptosporidiosis occurs among children and immunocompromised individuals [88–90]. In China, since the first report of human cryptosporidiosis in 1987, about 20 outbreaks have been reported. Cryptosporidiosis is endemic in at least 17 P/A/Ms with occurrence rates from 1.4 % to 10.4 %. Children and HIV/AIDS patients are more susceptible to infection [91]. A survey undertaken in 2008 showed that the prevalence of *Cryptosporidium* spp. in HIV/AIDS patients was 10.1 % (8/79), which was significantly higher than the 3.1 % (9/294) recorded in the control population [92]. High prevalence rates of *Cryptosporidium* in domestic animals have been reported in various areas. In addition to leading to significant economic losses, this is also a major risk factor for *Cryptosporidium* infections in humans [93, 94].

#### Giardiasis

Giardiasis is another common cause of acute and chronic diarrhea in humans and domestic animals. Among six known *Giardia* species, only *G. duodenalis* is responsible for infecting humans and most mammals. At least eight major genotypes (assemblages A–H) are known in this species. Of these, only assemblages A and B are zoonotic genotypes capable of infecting humans and other mammals [95]. Globally, about 200 million people suffer from symptomatic giardiasis. Children in developing countries are particularly at risk, with a prevalence of 20–30 %, as compared to the rate of 2–3 % in high-income countries [88]. In China, according to the first national survey conducted in 1992, giardiasis was endemic across the whole country with a mean infection rate of 2.5 %. The prevalence of giardiasis had declined significantly by the time the second national survey was undertaken in 2004 (e.g. from 3.85 % to 1.00 % in Zhejiang province) [47, 48, 91]. However, it was still common in children and in some underprivileged communities. *Giardia* oocysts were found in 32 of 762 school children (4.2 %) in Turpan area, Xinjiang Uygur in 2012 [96]. The prevalence of *G. duodenalis* infection in two rural villages of Anhui was 3.6 % (28/769) in 2008 [97].

#### Toxoplasmosis

Toxoplasmosis is caused by the obligate intracellular protozoan *Toxoplasma gondii*. Nearly one-third of the global human population is chronically infected. The disease also causes enormous economic losses to the livestock industry [98]. In China, the prevalence of *T. gondii* is relatively low compared with that in some European countries, but it appears to have increased over the past decade. The second national survey (2004) found a prevalence of 7.9 %, which was significantly higher than the 5.2 % prevalence found in the first national survey in 1992. Prevalence rates among some minority ethnic groups such as Miao (25.4 %), Buyi (25.3 %), and Mongol (17.1 %) were particularly high, perhaps as a consequence of these groups’ habit of eating raw or partially cooked meat [48, 99]. Seroepidemiological surveys in recent years showed that the prevalence of *T. gondii* is about 10–20 %, with a significantly higher prevalence rate in cancer patients (35.6 %) [100–102]. The prevalence of *T. gondii* in livestock is also high (about 15–31 %) [99]. The Chinese 1 (ToxoDB #9) genotype is the dominant genotype and exhibits a virulence similar to that of Type 1, which is predominant in North America and Europe [99].

### Emerging parasitic diseases of poverty

#### Angiostrongyliasis

Angiostrongyliasis, an emerging foodborne disease, is caused by *Angiostrongylus cantonensis*, which was first discovered in rats in Guangzhou in 1935 [103]. Now, it is endemic in Southeast Asia, Australia, the Caribbean, and the Pacific Islands. More than 2800 cases have been documented worldwide [104, 105]. In the past decades, the number of cases has sharply increased with several outbreaks reported. Between 1997 and 2011, several angiostrongyliasis outbreaks have been reported in China [106–108]. The largest outbreak occurred in Beijing city in 2006, which involved 160 cases [106]. Of them, 100 were hospitalized and four died. Investigators found that 75.1 % of those infected had eaten raw snails (*Pomacea canaliculata* and *Achatina fulica*), which was an important risk factor [106]. Food safety must be improved to interrupt the transmission of this parasitic disease.

### Perspectives for better control

Although China has made tremendous achievements in the prevention, control, and elimination of human parasitic diseases over 60 years of unremitting efforts, the burden of parasitic diseases is still great, especially in poor rural areas, due to poverty and inequalities in public services and environmental conditions [7–9]. Because China seeks to further reduce the burden of parasitic diseases, it is time to reflect on the progress made so far and what remains to be done. We now address the challenges and weaknesses in the control of parasitic diseases of poverty in the country, and outline the relevant strategies and measures to control them more effectively.

#### Poverty

China has undergone rapid and sustained economic development in the past three decades. However, economic development and consequent benefits are not evenly distributed across the vast geographical area of China. Western and central regions of the country are still less developed, the incidence of poverty is far greater, and health care is less accessible. Additionally, inadequate hygiene and sanitation, and a limited access to safe water have also contributed to the higher prevalence rates of parasitic diseases of poverty [109–112]. For example, temporal and spatial analysis carried out in Yunnan province indicated that poverty and malaria are closely linked: that is, low-income counties commonly have a high incidence of malaria [13]. Therefore, financial support for economic development in these counties is needed to increase the incomes and living standards of the poor. Secondly, an increase in the number of public health workers and hospital staff, especially in poor rural areas, is required. These workers need to be provided with specific training to equip them to deal with IDoPs. Thirdly, the basic healthcare coverage needs to be enhanced and the cost of health care must be lowered. Finally, provision of safe drinking water and related essential services must be increased in poor areas.

#### Environment

Rapid economic development has caused environmental damage, such as overgrazing and deforestation. Several large-scale environmental modifications, including the South-to-North Water Diversion Project (SNWTP) and the Three Gorges Dam Project (TGDP), have been implemented in 2002 and in 1994, respectively. These might aggravate the emergence and spread of parasitic diseases. For example, both SNWTP and TGDP can enhance the transmission of schistosomiasis and snail-borne trematodiases by enlarging snail habitats [113–115]. Global warming might affect the geographical distributions of vectors such as sandflies and mosquitoes [114]. Additionally, global warming might also affect the reproduction and extrinsic incubation period (EIP) of parasites. For example, the EIP of *P. falciparum* parasites are 26 days at 20 °C and 13 days at 25 °C [116]. Deforestation destroys wildlife habitats, thereby increasing interactions with humans and the transmission of zoonotic diseases, such as AE [81, 117, 118]. China must embrace the “One World, One Health” initiative to deal with health problems, including those pertaining to wild animals. Protection of the environment, as well as surveillance and responses to environmental and climate changes also need to be strengthened [1].

#### Globalization and urbanization

Globalization and urbanization have enhanced the mobility of people [119]. Every year, large numbers of Chinese people working abroad return home and many foreign laborers and travelers come to China. These people may introduce diseases to non-endemic regions of China. For example, in recent years, locally-transmitted *P. vivax* malaria has significantly decreased in prevalence but imported *P. vivax* malaria has sharply increased [23]. In the past three decades, China has undergone the largest-ever human migration with 260 million people moving from poor rural areas to the cities. This also has the potential to promote the spread of parasitic diseases of poverty and brings great challenges for the detection and control of epidemics [119]. Therefore, diagnosis and treatment of infectious diseases in migrants need to be strengthened.

#### Lifestyle and education

Over the past decades, the prevalence rates of foodborne parasitic diseases (e.g. angiostrongyliasis, clonorchiasis, and paragonimiasis) have increased significantly due to the habit of eating raw or undercooked fish, snails, and meat [8, 9]. Such dietary habits are traditional and remain common, such as the widely prevailing habit of eating raw fish in northeastern and southeastern China [53]. Traditional agriculture practices, such as the use of water buffaloes in the fields, also increase the possibility of parasitic infections [120]. Furthermore, some local religious beliefs limit the prevention and control of infectious diseases. In the echinococcosis-endemic west of China, killing of stray dogs (the definitive host of *E. granulosus*) is not possible due to religious beliefs [86]. Knowledge of, attitudes towards, and individual responses in light of this, especially by parents and influential community members, are crucial factors for preventing and controlling parasitic diseases. However, people in poor rural areas, especially children, have a low awareness of parasitic diseases [85, 121]. Health education packages, aiming to improve knowledge and awareness and change in hygiene behavior, are an effective way to prevent parasitic diseases [122]. Therefore, health education should be extensively promoted, especially in poor regions.

#### Diagnosis and drugs

Although low-intensity infections with parasites are often asymptomatic, they can damage human health and even cause death when left untreated. However, poor awareness of asymptomatic infections, as well as the current lack of sensitive and specific tools for early diagnosis, might accelerate the spread of parasitic diseases of poverty. For example, there is a lack of sensitive tools for the identification of asymptomatic malaria patients and for early diagnosis of echinococcosis [120]. Besides this, some of the available diagnostic tools are expensive, restricting their use in large-scale endemic surveys (i.e. diagnostic tests for *S. japonicum* infection) [120, 123]. Furthermore, prolonged use of certain drugs for treatment of some parasitic diseases might make parasites drug-resistant. An example of this is the emergence of chloroquine resistance in *P. falciparum* [24]. Therefore, in addition to rational use of antiparasitic drugs, a thorough understanding of the life cycles, biology, and gene functions of parasites is needed to develop sensitive and high-throughput diagnostic methods, as well as low-cost and effective drugs and vaccines.

#### Surveillance-response systems

Through persistent efforts, China achieved the elimination of lymphatic filariasis in 2007. Campaigns against other parasitic diseases such as schistosomiasis and leishmaniasis have moved from the control stage to the elimination stage, with only sporadic cases occurring [7, 124, 125]. Sparse data on some parasitic IDoPs are a key obstacle to controlling and eliminating these diseases. Effective and timely surveillance-response systems characterized by reliable information on the prevalence and spatiotemporal distribution of diseases, coupled with rapid-response capability to dispose of emerging threats, provide the final key step to achieving effective prevention, control, and elimination of IDoPs [126–128]. Due to the complexity and difficulty of controlling parasitic diseases of poverty, any surveillance-response system should include a range of elements. First, surveillance of parasitic infections in frontier regions and poor rural areas should be strengthened [129]. This includes surveillance in low-transmission areas where public health interventions have greatly reduced prevalence of infections. Second, surveillance data, including data from humans and animals, should be updated in a timely manner. Third, effective innovations and new technologies should be introduced into the surveillance-response systems, such as geographical information systems, global positioning systems, and geostatistical modeling. Finally, coordination between the control of parasitic diseases of poverty in humans and animals, as well as interdisciplinary and international cooperation must be strengthened due to the increase in the international transmission of infectious diseases [129].

## Conclusions

Significant progress has been made in the last 60 years to reduce the burden of parasitic diseases in China. However, considerable challenges remain, especially in poor rural areas. Fortunately, Chinese central and local governments have drawn up a range of control programs and implemented many integrated control strategies to prevent, control, and eliminate parasitic diseases of poverty. Some control strategies can serve as brilliant models for other developing countries, and China is willing to share its expertise and work with international partners to achieve the outcomes promoted by the “One World, One Health” initiative [1].

## Abbreviations

AE, alveolar echinococcosis; CE, cystic echinococcosis; CL, cutaneous leishmaniasis; DALYs, disability adjusted life years; DEC, diethylcarbamazine; EIP, extrinsic incubation period; IDoPs, infectious diseases of poverty; ML, mucosal leishmaniasis; P/A/Ms, provinces/autonomous regions/municipalities; SNWTP, South-to-North Water Diversion Project; STH, soil-transmitted helminth; TGDP, Three Gorges Dam Project; VL, visceral leishmaniasis

## Additional file

Additional file 1: Multilingual abstracts in the six official working languages of the United Nations. (PDF 500 kb)
