# Supplementary material for: Major parasitic diseases of poverty in mainland China: perspectives for better control
Source: Infect Dis Poverty. 2016 Aug 1;5:67. doi: 10.1186/s40249-016-0159-0 (PMC4967992; doi:10.1186/s40249-016-0159-0)
Supplement: Additional file 1: — Multilingual abstracts in the six official working languages of the United Nations. (PDF 500 kb) [file 40249_2016_159_MOESM1_ESM.pdf]

Translation of the abstract into the six official working languages of the United Nations

الأمراض الطفيلية المتعلقة بالفقر الشائعة في الأراضي الصينية :أفكار لسيطرة أكبر.

جبن-لي وانغ، تينغ-تينغ لي، سي-يانغ هوانغ، واي كونغ، سينغ-كوان زو.

خلاصة:
إن تقدماً ملحوظاً قد لاقى نجاحاً باهراً في منع الأمراض و السيطرة عليها و حتى إزالة الأمراض الطفيلية البشرية في الصين كليّاً في الستين ٦٠ سنة الأخيرة. رغم ذلك، الأمراض الطفيلية المتعلقة بالفقر تبقى مسبباً رئيساً لتفشي الأمراض و ازدياد الوفيات و تلقي بأعباء اقتصادية هائلة على عاتق المجتمعات.
في هذا المقال، نراجع معدلات تفشي الأمراض، التوزعات الجغرافية، الخصائص الوبائية، عوامل الخطر و المظاهر السريرية للأمراض الطفيلية المتعلقة بالفقر المدرجة في المشكلة الأولى في مجلة ”أمراض معدية عن الفقر” في ٢٥ تشرين الأول لعام ٢٠١٢. نحن نهتم أيضاً بذكر التحديات التي تواجه التغلب على هذه الأمراض و ابتكار مقترحات لحلول أفضل.

Translated from English version into Arabic by Marcel Alied, through

TRANSLATORS
WITHOUT BORDERS

中国大陆与贫穷有关的主要寄生虫病：研究进展及有效的防控前景

Jin-Lei Wang, Ting-Ting Li, Si-Yang Huang, Wei Cong, Xing-Quan Zhu

摘要：
新中国成立以来，中国在寄生虫病的预防、控制、消除方面取到了巨大的成就，但是与贫穷有关的寄生虫病仍然严重危害着人类和家畜的生命与健康，造成严重经济损失，制约着我国的经济发展。本文概述了《Infectious Diseases of Poverty》首刊列出的、中国大陆与贫穷有关的主要寄生虫病，介绍了它们的流行趋势、地域分布、风险因素及临床特征，阐述了在防控这些与贫穷有关的寄生虫病方面的挑战，并提出了正确地应对这些挑战的措施。

Translated from English version into Chinese by Jin-Lei Wang

Maladies parasitaires majeures liées à la pauvreté en Chine continentale : perspectives pour un meilleur contrôle

Jin-Lei Wang, Ting-Ting Li, Si-Yang Huang, Wei Cong, et Xing-Quan Zhu

Abstract
Des progrès significatifs ont été réalisés en prévention, contrôle et élimination des maladies parasitaires humaines en Chine, ces 60 dernières années. Toutefois, les maladies parasitaires liées à la pauvreté restent des causes majeures de morbidité et de mortalité, et elles représentent des coûts économiques énormes pour la société.
Dans cet article, nous examinons les taux de prévalence, les distributions géographiques, les caractéristiques épidémiques, les facteurs de risque et les manifestations cliniques des maladies parasitaires liées à la pauvreté énumérées dans le premier numéro du journal *Infectious Diseases of Poverty* le 25 octobre 2012. Nous abordons aussi les défis liés au contrôle des maladies parasitaires liées à la pauvreté et faisons des suggestions pour un meilleur contrôle.

Translated from English version into French by Jacek Sierakowski, through

Основные паразитарные болезни бедного населения материковой части Китая: перспективы контроля

Цзинь-Лэй Ван, Тин-Тин Ли, Сы-Ян Хуан, Вэй Цун и Син-Цюань Чжу

Аннотация

За последние 60 лет в Китае был достигнут значительный прогресс в профилактике, контроле и искоренении паразитарных болезней. Несмотря на это, паразитарные болезни бедных остаются основной причиной заболеваемости и смертности среди населения, а также влекут за собой значительные экономические издержки для общества.

В данной статье рассматриваются коэффициенты распространенности заболевания, географическое распределение, эпидемиологические характеристики, факторы риска, а также клинические проявления паразитарных болезней бедного населения, которые приведены в первом издании журнала «*Infectious Diseases of Poverty*» от 25 октября 2012 г. В ней также отражены проблемы по осуществлению контроля за паразитарными болезнями бедного населения и приводятся предложения по контролю за ними.

Translated from English version into Russian by Yuliya Maiseyenka, through

Principales enfermedades parasitarias de la pobreza en China continental: perspectivas para un mejor control

Jin-Lei Wang, Ting-Ting Li, Si-Yang Huang, Wei Cong, and Xing-Quan Zhu

Resumen

La prevención, el control y la eliminación de enfermedades parasitarias en humanos en China han progresado de manera significativa en los últimos 60 años. Sin embargo, las enfermedades parasitarias de la pobreza todavía representan la mayor causa de morbilidad y mortalidad, e infligen enormes costos económicos en las sociedades.

En este artículo, revisamos las tasas de prevalencia, las distribuciones geográficas, las características epidemiológicas, los factores de riesgo, y las manifestaciones clínicas de las enfermedades parasitarias de la pobreza, mencionadas en el primer número de la publicación *Enfermedades Infecciosas de la Pobreza* el 25 de octubre de 2012. También nos referimos a los desafíos para lograr controlar las enfermedades parasitarias de la pobreza y presentamos algunas sugerencias para lograr un mejor control.

Translated from English version into Spanish by Mpgorgone, through
